# The effect of choice and reward on motivation and performance: an eye-tracking study

**DOI:** 10.3389/fpsyg.2026.1905792

**Published:** 2026-08-13

**Authors:** Rosa Hendijani, Piers Steel

**Affiliations:** 1 Faculty of Management, University of Tehran, Tehran, Iran; 2 Haskayne School of Business, University of Calgary, Calgary, AB, Canada

**Keywords:** choice, overall motivation, performance, performance-contingent rewards, visual attention

## Abstract

**Introduction:**

Choice and reward are two prevailing motivational mechanisms. However, previous studies have found differing results regarding their effects. According to the literature, several factors including task and contextual characteristics can influence their effects. In line with motivational congruence theory (MCT), this study examines the effect of these motivational mechanisms in a controlling context, characterized by an attention-demanding memory test.

**Methods:**

This study uses a lab experiment to examine the direct effect of performance-contingent rewards and choice and their indirect effect through visual attention on overall motivation and performance. In total, 270 individuals participated in a laboratory experiment with a 3 (choice: no-choice, choice, and control) × 3 (performance-contingent reward: control, non-salient, and salient) factorial design. Participants were randomly assigned to one of the experimental conditions. Choice and reward were manipulated based on the literature. Visual attention was measured using an eye-tracking technique. Overall motivation and performance were measured by time spent on the memory test and number of correct answers in the test.

**Results:**

The results support the hypotheses. Both salient and non-salient rewards increased overall motivation and performance, whereas choice had no significant effect. In addition, visual attention towards the reward-related stimuli mediated the effect of both reward types on overall motivation and performance.

**Discussion:**

The results align with MCT's predictions and elaborate on the cognitive mechanisms (i.e., visual attention) that underlie motivation and performance. They highlight the cognitive and behavioral effects of non-salient and salient rewards in strengthening motivation and performance in a controlling context.

## 1 Introduction

Choice (Flowerday and Schraw, 2000; Gureckis and Markant, 2012a; Patall et al., 2010) and reward (Garbers and Konradt, 2014; Cerasoli et al., 2014; Van Herck et al., 2010) are two motivational mechanisms that are widely utilized to enhance motivation and performance (Chiew and Adcock, 2019). Despite widespread use, their administration has not been consistently beneficial (Fall and Roussel, 2014; Katz and Assor, 2007; Overskeid and Svartdal, 1996; Patall et al., 2014; Reeve et al., 2003). Previous studies have shown that several factors, including task characteristics (Hendijani and Steel, 2022) and context (Koh, 2020; Kuvaas et al., 2016; Savani et al., 2010; Thomas and Mueller, 2017) can influence their effects. However, previous studies have mainly examined these effects from a behavioral perspective. However, these studies have mainly focused on the effect of reward and choice on behavioral outcomes of overall motivation and performance. This study expands the current literature by examining the cognitive foundations (i.e., visual attention toward the reward-related stimuli) underlying these behavioral outcomes (i.e., overall motivation and performance). Using a lab experiment with eye-tracking assessment, this study investigates the effect of salient and non-salient performance-contingent rewards and choice on visual attention, overall motivation, and performance in a controlling context.

To clarify the main constructs used in the study, we define them here. Reward refers to an external motivational mechanism (e.g., money, bonus) that encourages one toward a specific action (Steel and Weinhardt, 2018; Schwartz, 1989). Reward salience is a characteristic through which the reward becomes perceptually prominent and conspicuous during task engagement (Hewett and Conway, 2016; Tran and Howard, 2026). Choice refers to the provision of different options to an individual so that she/he can select one of them (Patall et al., 2010). Overall motivation is the combination of different motivational mechanisms that creates a total force toward action (Hendijani et al., 2016). Performance is the behavioral outcome or an action that one produces in the end (Folan et al., 2007; Sonnentag and Frese, 2002). A controlling context incorporates environmental and task-related elements, such as attention demand, strict rules, time limit, and lack of intervention and participation that produce perceptions of control rather than autonomy in the mind of the individual (Gureckis and Markant, 2012a; Hendijani and Steel, 2024).

Mainstream theories in motivation present differing perspectives regarding the effect of performance-contingent rewards and choice on overall motivation and performance (Hendijani and Steel, 2020; Kim et al., 2022). Behavioral theories in psychology and classic economics consider extrinsic motivation (e.g., reward) as the main mechanism for motivation and performance improvement (Eisenberger and Cameron, 1996; Skinner, 1953; Steel and König, 2006; Steel and Weinhardt, 2018; Vroom, 1964). Extrinsic motivation refers to the type of mechanism, such as monetary reward and positive feedback, and is separate from the innate nature of the task but still reinforces behavior (Deci and Ryan, 2000; Kuvaas et al., 2016). Based on these theories, rewards, especially performance-contingent ones, can reinforce motivation and performance (Porter and Lawler, 1968). Cognitive evaluation theory (CET) and self-determination theory (SDT), on the other hand, focus on intrinsic motivational mechanisms (Deci et al., 1999) and give a higher weight to them compared to extrinsic ones (Hendijani and Steel, 2024; Ryan and Deci, 2022). Intrinsic motivation, as defined by these theories, is the inherent desire toward doing an activity without any attached extrinsic outcomes (Ryan and Deci, 2022). These theories highlight autonomy as one of the underlying pillars of intrinsic motivation. Accordingly, motivational mechanisms that reinforce autonomy can increase intrinsic motivation. Choice is one of the autonomy-reinforcing mechanisms (Patall et al., 2008). On the other hand, motivational mechanisms that are controlling undermine intrinsic motivation. According to these theories, performance-contingent rewards are controlling and thus diminish intrinsic motivation.

Motivational congruence theory (MCT) adds to these theoretical streams by giving equal weight to intrinsic and extrinsic motivations (Hendijani and Steel, 2024). Accordingly, both intrinsic and extrinsic motivational mechanisms are posited to positively influence overall motivation and performance. However, the simultaneous and interactive effect of these motivational mechanisms depends on their congruence with one another and the context within which they are administered (Hendijani and Steel, 2024; Hendijani, 2024a,b; Saini et al., 2025; Tran and Howard, 2026). According to MCT, overall motivation emerges from the integration of intrinsic motivation, extrinsic motivation, and the context, which in combination gives rise to behavior (Hendijani and Steel, 2024). Previous empirical studies supported MCT's predictions by examining the effect of reward and choice with different task types (e.g., cognitive and attention-demanding tasks) and in different contexts (e.g., autonomy-supportive and controlling contexts).

This study contributes to the field from both behavioral and cognitive perspectives. From a behavioral perspective, it provides further evidence for MCT's predictions regarding the effect of these two motivational mechanisms on overall motivation and performance in a controlling context. The type of task used in this study is an attention-demanding explicit memory test. The high level of attentional demand makes the context controlling. Besides, other contextual and task characteristics contribute to the controlling nature of the task environment. These include a pre-determined sequence of activities, not allowing participants to go back during the test, having a fixed time limit, and automatically ending the test after the fixed time interval (Amabile et al., 1976; Kuvaas et al., 2017; Ryan et al., 1983). Similar to the findings of an earlier study (Hendijani and Steel, 2022), the behavioral results indicate that choice does not have a significant effect on overall motivation and performance in this context. In contrast, compared to the control condition, salient and non-salient rewards improve overall motivation and performance. From a cognitive perspective, the study integrates the cognitive and behavioral underpinnings of motivation and performance by also assessing visual attention toward the reward-related stimuli. Eye tracking is an advanced technique that captures the cognitive foundations of motivation and behavior by objectively capturing one's involuntary reactions to motivational stimuli (Mark et al., 2024; Popa et al., 2015; Samadani, 2015). Addressing the call for an integration of behavioral and cognitive approaches (King and Fryer, 2024; Pekrun, 2024), current study deepens the predictions of behavioral theories by shedding light on the cognitive mechanisms underlying overall motivation and performance.

## 2 Literature review

Motivational mechanisms have their own distinct effects when administered separately or in combination with one another (Woolley and Fishbach, 2018), the specifics of which has been a matter of debate (Hendijani et al., 2016). This study focuses on two main motivational types: performance-contingent reward and choice. Previous theoretical streams have had contradictory predictions regarding the effect of these two motivational mechanisms on overall motivation and performance (Hendijani and Steel, 2020, 2022). In the next sections, we review the literature related to each of these motivational mechanisms and produce the research hypotheses for the current study.

### 2.1 Effects of choice on overall motivation and performance

By definition, choice refers to the opportunity of selecting between different options (Merriam-Webster, 2024) and is an embodiment of autonomy and free will (Patall et al., 2008; Patall and Hooper, 2019; Ryan and Deci, 2000). Choice can promote feelings of “personal causation” (DeCharms, 1968, p. 273) and increase perseverance in achieving the desired outcomes (Zuckerman et al., 1978). In addition, it can result in higher levels of interest and satisfaction with what were the consequences of the choice (Cordova and Lepper, 1996). According to CET and SDT, choice can satisfy the need for autonomy, one of the basic needs that underlie intrinsic motivation. Thus, the provision of choice can improve intrinsic motivation and performance, with numerous empirical studies providing support for the positive effects of choice on the former (Iyengar and Lepper, 1999; Zuckerman et al., 1978) and the latter (Cordova and Lepper, 1996).

Despite theoretical elaboration and empirical support for the positive effect of choice, there is also evidence for a negative or null effect on motivation and performance (Carlisle et al., 2022; Patall and Hooper, 2019; Reeve et al., 2003). In a series of studies, Flowerday and Schraw (2003) found that giving participants the opportunity to choose between working on an essay or playing a word puzzle had no significant effect on engagement and performance compared to the no choice condition. In a follow-up study, they found that participants who were able to choose the pace of their own task exerted less effort and performed worse compared to those whose pace was determined by the experimenter. In other studies, participants in the choice condition showed lower performance quality compared to the participants in the no-choice condition (Flowerday et al., 2004).

Consistent with the mixed results of previous studies, MCT posits that the effect of choice depends on its congruence/incongruence with the context and existing motivational mechanisms (Hendijani and Steel, 2022, 2024). Specifically, provision of choice in combination in an autonomy-supportive context can create positive effects. On the other hand, the provision of choice in a controlling context can produce null or negative effects depending on the level of perceived control (Hendijani and Steel, 2022, 2024). Investigations along these lines produce consistent results. In one study, researchers found that provision of choice in an autonomy-supportive task context resulted in positive effects of choice on overall motivation and performance (Hendijani and Steel, 2020). In another, the researchers found that the provision of choice in a controlling task context produced no effect for choice on overall motivation and performance (Hendijani and Steel, 2022).

### 2.2 Effect of performance-contingent rewards on overall motivation and performance

The effect of performance-contingent reward on motivation and performance has produced longstanding and controversial debate in the motivation literature. Reinforcing theories, including expectancy-value and reinforcement theories posit that external rewards are the main types of motivational mechanisms (Hendijani and Steel, 2024). According to these theories, external rewards, especially performance-contingent ones, can reinforce motivation and performance by creating a direct and transparent causal link between the reward and the task in the mind of the individual (Porter and Lawler, 1968). Many empirical studies (Eisenberger and Aselage, 2009; Lacetera et al., 2012; Hendijani et al., 2016) and meta-analytical reviews (Byron and Khazanchi, 2012; Garbers and Konradt, 2014) have supported these explications.

Undermining theories, including CET (Deci and Ryan, 1985) and SDT (Ryan and Deci, 2000, 2019, 2020), give higher priority to intrinsic motivation compared to extrinsic motivation. According to these theories, intrinsic motivation refers to inner interest toward an activity without the expectation of any appended outcome. Autonomy is one of the psychological needs that underlie intrinsic motivation. These theories argue that external rewards, especially performance-contingent ones, are coercive and controlling in nature and thus undermine autonomy and intrinsic motivation (Deci et al., 1999). Empirical studies and meta-analytical reviews have provided support for the undermining effect of performance-contingent rewards on intrinsic motivation (Deci et al., 1999). However, the undermining effect is not universal (Woolley and Fishbach, 2018). Several factors exist in the design and implementation of these studies that limit the interpretation and generalization of their results. High levels of reward salience and the use of games instead of realistic task types are among these factors (Hendijani et al., 2016). Furthermore, the undermining effects are usually temporary and occur in a short period after the main experimental session (i.e., free choice period), when the reward is suddenly removed and is no longer present (Deci et al., 1999; Goswami and Urminsky, 2017; Hendijani et al., 2016).

As a third stream, contingency theories focus on the causal attribution by the individual rather than the actual cause of the behavior. These theories posit that the negative effect of performance-contingent rewards on intrinsic motivation can be due to the salience of the reward rather than its existence and contingency on performance. Salient reward refers to the type of reward that is conspicuous or prominent during task engagement and accomplishment (Hewett and Conway, 2016; Ross, 1975; Taylor and Fiske, 1978). In contrast, non-salient reward is inconspicuous or lacks perceptual prominence during task accomplishment (Eisenberger and Selbst, 1994). According to these theories, as the reward becomes salient, individuals attribute their motivation and performance to the reward and lose motivation once the reward is removed (Ferrin and Dirks, 2003).

MCT resolves these discrepancies in the existing theoretical streams by going beyond the traditional Manichean or dualistic approach in the motivation literature by giving equal weight to both intrinsic and extrinsic motivational mechanisms (Hendijani and Steel, 2024). In addition, MCT adds the role of context to the effect of different motivational mechanisms. According to this theory, the effect of a motivational mechanism depends on its congruence/incongruence with other motivational mechanisms and the context within which they are applied (Hendijani, 2021). In an autonomy-supportive context, the provision of non-salient rewards and choice can reinforce overall motivation and performance. In a controlling context, the provision of salient rewards and lack of choice can improve overall motivation and performance.

Previous empirical studies have supported these predictions. One experimental study examined the effect of reward (2 levels: salient and non-salient performance-contingent rewards) and choice (2 levels: choice, no-choice) on overall motivation and performance in an autonomy-supportive context (Hendijani and Steel, 2020). The results indicated that non-salient reward and choice positively interacted and increased overall motivation and performance. A similar effect was found for the salient reward and no-choice combination. Another study examined the effect of reward salience and choice in a controlling context (Hendijani and Steel, 2022). The results indicated that choice had no significant effect on overall motivation and performance. However, both non-salient and salient rewards improved overall motivation and performance.

### 2.3 Cognitive mechanisms underlying the reward's effect

One of the main issues in the motivation literature is to establish the cognitive mechanisms underlying the reward's effect (Arsalidou et al., 2020). This addresses the type of cognitive mechanisms that mediate the effect of rewards on overall motivation and performance. Visual attention is one of the underlying cognitive mechanisms that is widely studied in the reward literature (Brielmann and Spering, 2015; McCoy and Theeuwes, 2016; Orquin and Loose, 2013).

The mediating effect of visual attention depends on two linkages: one is between the reward and visual attention and the other is between visual attention and motivation and performance. Regarding the first link (i.e., reward-visual attention), previous studies have shown that reward can influence visual sensitivity and attention toward the reward-related stimuli (Anderson et al., 2011; Brielmann and Spering, 2015; Hickey et al., 2010; Pascucci and Turatto, 2013). The presence of reward, as shown in these studies, results in a shift of focus toward the sign or object that is related to reward (Schütz et al., 2012). These studies are often designed in the form of a laboratory-based binary choice task in which two visual stimuli appear on the screen. One of them is associated with a reward while the other one is not. Participants should select a location by moving their eyes as fast as possible toward the related location. The results generally indicate that the individual's eyes tend to move faster and more accurately toward the rewarded compared to the non-rewarded stimuli (Hayhoe and Rothkopf, 2011; Sohn and Lee, 2006; Theeuwes and Belopolsky, 2012). Visual attention improves with increase in the reward's value (Shohamy and Adcock, 2010) or salience (Madan and Spetch, 2012).

Regarding the second link (i.e., between visual attention and motivation and performance), previous studies have shown that visual attention influences motivation (Issa et al., 2015) and behavior (Cavanagh et al., 2014; McCoy and Theeuwes, 2016). In some studies, the researchers found that biasing visual attention was related to motivation (Issa et al., 2015). In addition, manipulation of visual attention causally affected preference (Shimojo et al., 2003). In other studies, the results showed that visual attention in the form of fixation count predicted preference and behavioral choice (Armel et al., 2008; Glaholt et al., 2009; Orquin and Loose, 2013; Shimojo et al., 2003). Higher levels of visual fixation predict attraction and pursuit toward a specific option compared to other options.

## 3 Research hypotheses

This study examines two sets of hypotheses, including behavioral and cognitive ones. Behavioral hypotheses focus on the effect of performance-contingent rewards and choice on overall motivation and performance. Cognitive hypotheses focus on the cognitive mechanisms (i.e., visual attention) that mediate this relationship. In the next two sections, these hypotheses are elaborated.

### 3.1 Behavioral hypotheses

This study examines the effect of performance-contingent reward and choice on overall motivation and performance in a controlling context. Performance-contingent reward is manipulated in salient and non-salient forms and is contrasted with the control condition. In line with MCT's predictions (Hendijani, 2024a,b) and the results of previous empirical studies (Hendijani and Steel, 2022), we predict that choice does not have any effect on overall motivation and performance in a controlling context. Choice is an external mechanism that reinforces the perception of autonomy (Patall, 2013). A controlling context acts in an opposite direction and forces the individual to focus attention and exert behavior toward a specified action. Previous empirical studies have shown that controlling environments characterized by high level of task attentional demand (Hendijani and Steel, 2022), time and performance pressure (Amabile et al., 1976; Muraven et al., 2008), and the restriction of choice (Moller et al., 2006) can negatively affect the perceptions of autonomy and cancel or reverse the effect of choice on motivation and performance outcomes. The insignificant effect of choice on motivation and performance has been supported in different tasks and across different contexts (Flowerday et al., 2004; Flowerday and Schraw, 2003; Hendijani and Steel, 2022; Mozgalina, 2015; Reeve et al., 2003). Thus, choice provision is predicted to have a minor effect under controlling conditions and we have the following hypotheses:

**Hypotheses 1a/1b**. Choice has a small effect on overall motivation/performance in a controlling context.

In contrast with choice provision, performance-contingent rewards are in congruence with controlling environments. According to self-determination and cognitive evaluation theories, these types of motivational mechanisms are controlling in nature (Ryan and Deci, 2022). Previous empirical studies have shown that the provision of performance-contingent rewards in controlling contexts characterized by high levels of time pressure and performance monitoring can reinforce a controlled type of motivation (Hendijani and Steel, 2022; Kuvaas et al., 2016; Weibel, 2010). Thus, performance-contingent rewards can positively influence overall motivation and performance in a controlling context and we have the following hypotheses.

**Hypotheses 2a/2b**. Performance-contingent reward has a positive effect on overall motivation/performance in a controlling context.

### 3.2 Mediating effects of visual attention

One of the aims of this study is to examine the cognitive mechanisms that mediate the effect of reward on overall motivation and performance. This study focuses on visual attention as a critical cognitive mechanism that mediates these effects. Previous empirical studies have shown that rewards increase visual attention toward the reward-related stimuli (Hayhoe and Rothkopf, 2011; Hickey et al.,2010; Schütz et al., 2012). In addition, visual attention positively influences overall motivation (Issa et al., 2015; Shimojo et al., 2003) and performance (Armel et al., 2008; Glaholt et al., 2009; McCoy and Theeuwes, 2016; Orquin and Loose, 2013). Considering these, we have the following hypotheses:

**Hypotheses 3a/3b**. Visual attention to reward-related stimuli mediates the effect of performance-contingent reward on overall motivation/performance.

## 4 Research method

### 4.1 Experimental design

To test the research hypotheses, a 3 (reward: control, non-salient, salient) × 3 (choice: control, no-choice, choice) laboratory experiment was designed and conducted.

The study received ethics approval from the Research Ethics Boards at two universities in Iran and Canada. Participants were students and graduates from different universities inside the city of Tehran, Iran. To attract participation, recruitment notices were distributed across university campuses, students' social networks, and classes in several universities in the city of Tehran. In the recruitment notice, information was provided regarding different aspects of the experiment, including the required amount of time (approximately 1 h), voluntary participation and withdrawal, and the use of eye-tracking equipment in the study.

### 4.2 Experimental procedure

The study was conducted in a neuroscience research lab at a large Iranian university. All the study material were in Persian language, the participants' primary language. Here are the steps of the experimental procedure. First, upon arrival at the lab, the candidates were asked to carefully read their consent forms and given time to ask questions regarding the experimental procedure. Second, they signed the consent form and returned it to the experimenter if they were willing to proceed with the study. Third, they were randomly assigned to one of the nine experimental conditions, whereupon the main experimental session started. Fourth, participants answered several questions related to their general ability, interest, and confidence in doing memory tests. Since the experimental task was a memory test, these questions were used to collect information and control for the effect of individual characteristics on performance in these test types (Hendijani et al., 2016). Fifth, the manipulation for reward and choice were conducted, respectively. These manipulations are explained in the “Choice and Reward Manipulations” section.

Sixth, after the manipulation section, participants completed a memory test on the computer. During the memory test, participants' eye movements were tracked. Participants were requested to sit at a distance of 50 cm from a 23-inch computer monitor with a 1024 × 768 pixel resolution and remain there during the experiment. The eye-tracking device used was a Tobii X2-60 eye-tracker (Tobii Technology, Stockholm, Sweden), which is unobtrusive being attached to bottom of the computer screen.

**Figure 1:**
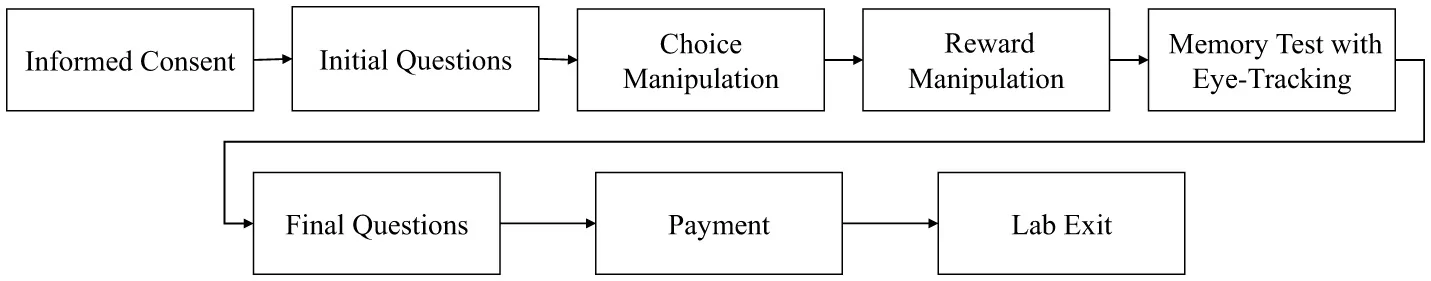
Study procedure.

Seventh, after the memory test, there were a set of questions related to choice and reward manipulation checks and demographics. Finally, participants were guided to another room where they received payments depending on their reward conditions and left the lab. The study procedure is presented in Figure 1.

### 4.3 Memory test characteristics

The task used in this study was a memory test. It was designed based on the standard approach used in previous studies (e.g., Graf et al., 1985; Hendijani and Steel, 2022). The test consisted of seven trials and each trial consisted of 12 words that appeared one by one on the screen. The words in each trial were randomly selected from different word categories. The categories were based on the schema developed by Battig and Montague (1969). In total, there are 16 categories, such as birds, fruits, vegetables, colors, animals, and musical instruments. The words were presented to all participants in the same order. Following the protocol used in previous memory tests, we selected the words that did not rank as the 10 most frequently mentioned, but were recalled by approximately 10 people out of 400 people (i.e., Battig and Montague, 1969; Mulligan and Hartman, 1996; Rappold and Hashtroudi, 1991). Of note, the information related to the frequencies and recall levels of the words were calculated based on previous studies conducted in Persian language (Miller and Aghajanian-Stewart, 2018). The average ranking of the selected words in each of the 16 mentioned categories was 22 with a range of 11–40. Participants were instructed to click the “Next” button to go to the next word or next section. After each trial of 12 words, participants were directed toward a specific page, where they could type the words they recalled. Once they were done with the recall phase, they moved to the next trial.

### 4.4 Experimental context

The experiment had several characteristics, which resulted in a controlling context. First, the utilized memory test is of an *explicit* type. Explicit memory tests need conscious and purposeful recollection of prior events. In contrast, *implicit* memory tests do not require any conscious and intentional remembering of a prior event (Mulligan, 1997). Compared to implicit memory tests or free recall tests, explicit ones require high levels of concentration and attention during the word presentation section (Fisk and Schneider, 1984; Mulligan, 1998). Second, participants need mindful information processing during the recall section of these test types. Demand for high levels of attention and mindfulness during task accomplishment makes these memory tests naturally controlling (Hendijani and Steel, 2022; Verhaeghen, 2021). Third, participants were presented with a series of pre-determined and pre-ordered set of items that were selected by the experimenter and presented to them. They had no control over the order, sequence, and categories of these items and the way they should interact with them. This decreased the level of self-direction while performing the task and forced the participants to follow the order and rules of a pre-designed test (Gureckis and Markant, 2012a). Fourth, participants were not allowed to go back and check any of the previously presented items and had to proceed with the order that was incorporated by the experimenter. Fifth, although the task was self-paced, the total amount of time was limited and the participants were given 1 h to finish the whole memory test. Sixth, if they did not finish the test within the pre-determined interval, the system automatically closed the memory test section.

In total, the attention-demanding and focused nature of the task (i.e., explicit memory test) along with other experimental characteristics (e.g., pre-determined sequence of words, not allowing backward movement, and definite time limit) created a condition that lacked self-direction and autonomy, resulting in a perceptually controlling situation (Gureckis and Markant, 2012a).

It is important to distinguish between the controlling context and the condition of no-choice, which is used in the choice manipulation in this study. The context is the environment where diverse motivational mechanisms interact (Hendijani, 2024a). A controlling context comprises of particular characteristics of the task or the environment that force the individual toward the attainment of a particular goal (Weibel et al., 2014). Such conditions act in the opposite direction of autonomy (Walton, 1985). Consequently, task or environmental characteristics such as high level of structure, workload (Kuvaas et al., 2017), attention demand (Ackerman, 1987; Mulligan, 1997), time pressure (Amabile et al., 1976; Muraven et al., 2008; Ryan and Deci, 2000), and constant feedback and monitoring (Enzle and Anderson, 1993) are among the factors that can make the task context controlling. Choice provision, on the other hand, is a specific motivational mechanism used to improve feelings of perceived autonomy and boost overall motivation and performance (Patall et al., 2008). Thus, a no-choice condition encompasses a situation where the individuals have been denied a certain type of choice that has been provided to individuals in the choice condition.

### 4.5 Choice and reward manipulations

Choice manipulation was at three levels: choice, no-choice, and control. The protocol used for the choice and no-choice conditions were similar to the previous studies conducted by Hendijani and Steel (2020), 2022). In the choice condition, participants read an explanation regarding two memory tests (i.e., Test A and Test B) and were asked to choose between them. The two tests were highly similar and had minor differences. The difference was in the order of presented words in the last set in the two memory tests. Using similar task types allowed us to address the common problem of conflating the effect of choice from task type or interest (Hendijani and Steel, 2022). If the task alternatives were not similar, there was a possibility that the effect of choice manipulation on overall motivation and performance is confounded with factors, such as interest, confidence, familiarity, or competence. As a result, we could not readily separate the effect of choice from these confounding variables.

In the no-choice condition, participants were told that they had no choice regarding the test type and should complete a test that was assigned to them by the experimenter. To further emphasize a lack of autonomy, they were told that participants in another group were given the choice regarding the type of test that they wanted to take. The explanation was mentioned in a note that appeared on the screen at the beginning of the memory test. The words related to the lack of choice were written with bold fonts and were underlined to emphasize the manipulation. In the control condition, participants were simply asked to complete a memory test without any further explanation.

**Figure 2:**
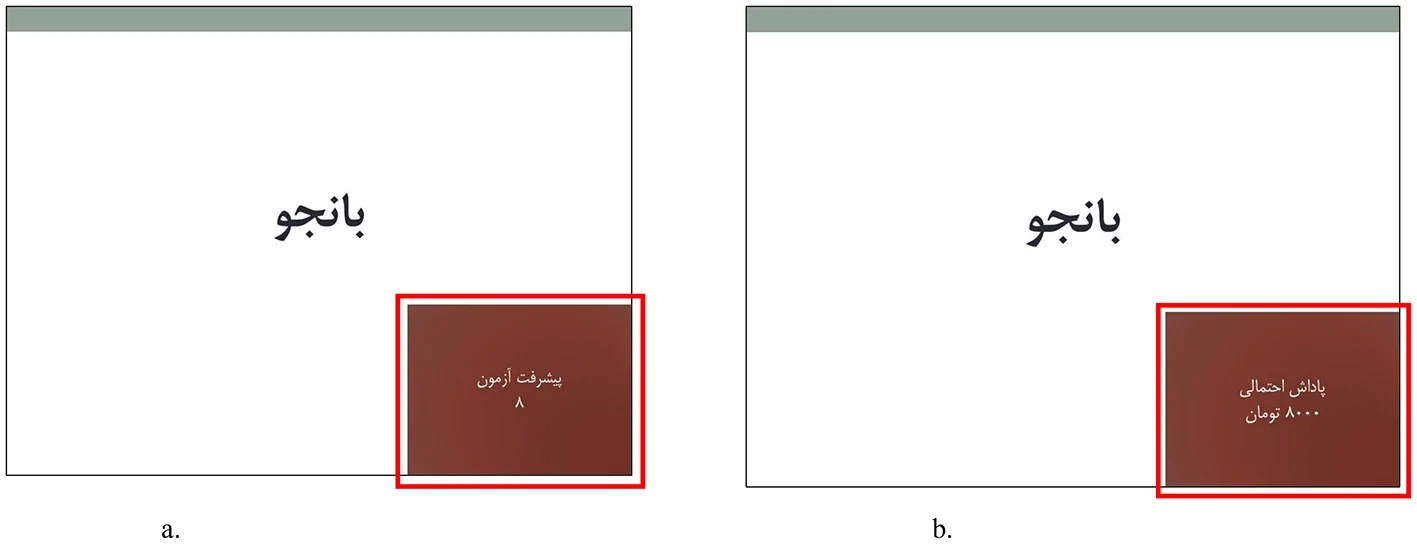
**(a)** Sample picture of a word in non-salient and control reward conditions. **(b)** Sample picture of a word in the salient reward condition.

**Figure 3:**
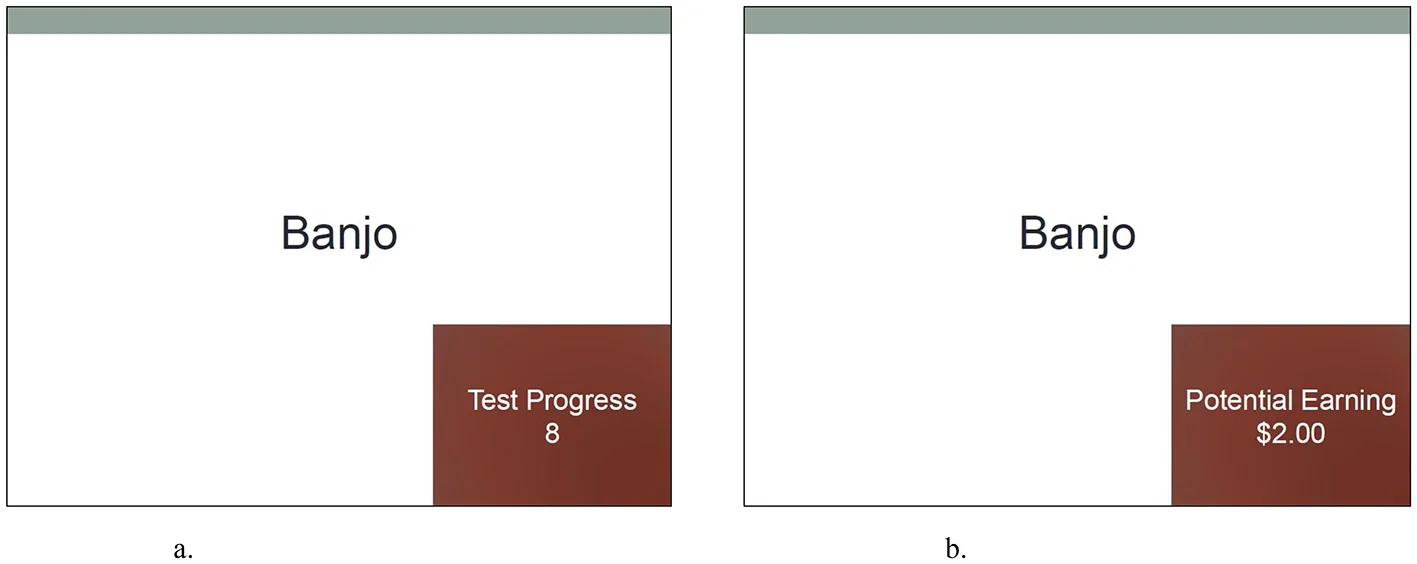
**(a)** Sample picture of a word in non-salient and control reward conditions (Translated version). **(b)** Sample picture of a word in the salient reward condition (Translated version).

Reward manipulation was conducted with three levels of salient reward, non-salient reward, and control conditions. The manipulation followed previous studies conducted by Hendijani and Steel (2020; 2022). Participants in all conditions received a fixed pay of 500,000 IRR (approximately $1 US) as their participation fee. The pay was set at a competitive level for the local economy, based on the approximate hourly pay rate for university students. In addition to the participation fee, participants in salient and non-salient conditions received a payment of 10,000 IRR (20 cents US) for each correctly recalled word in a set of memory tests. Participants in both non-salient and salient conditions were informed of the performance-contingent reward at the beginning of the memory test. To make the reward more prominent and conspicuous during the memory test, participants in the salient conditions saw a large amount of money at the beginning of the memory test. Below the money photo, this statement was written: “You are able to earn a large amount of money if you perform well in this test.” In addition, a number called potential earning was placed in a brown box at the bottom right-hand side of the screen during the memory test. Potential earning was equal to the amount of money that participants could earn if they had correctly recalled all the words up to that point. For example, the potential earning amount appearing on the screen for the first and eleventh words in the first memory test were equal to 10,000 IRR and 110,000 IRR, respectively. To keep the same level of distraction in non-salient and control condition, participants in these conditions received numerical test progress instead of the potential earning in the brown box in the bottom right hand side corner, which is informationally but not motivationally equivalent. Figures 2a, b present a sample screen of the memory test in the three reward conditions. For clarification, Figures 3a, b provide the English translated version of the same screens. As can be seen the information related to test progress and potential earning are displayed in a brown box at the bottom right hand side of the screen.

### 4.6 Study variables

Choice and reward are the independent variables and overall motivation and performance are the dependent variables. Visual attention toward the reward/progress related information are the mediating variables. It was assessed by use of several measures, including time to first fixation (TFF), fixation duration (FD), and fixation count (FC) toward the area that presented information related to the potential reward or test progress on the screen. In order to assess these measures, an area of interest (AOI) was captured on each of the word screens, which presented the potential earning in the reward condition and test progress in the non-salient and control conditions. This area was the brown box at the bottom right hand side of the screen. The AOIs are captured in Figures 2a, b, as the areas with a red rectangle around them. Finally, an average value was calculated for all pictures in the memory test to reach one single score for each of the three measures of TFF, FD, and FC. These measures are frequently used for assessing visual attention and allow us to capture visual attention from several dimensions.

Choice is a categorical variable and has three levels, including control, no-choice, and choice conditions. Reward is a categorical variable and has three levels of salient, non-salient and control. Overall motivation is measured by the amount of time spent on the experimental task (i.e., a memory test). This measure is commonly used as a behavioral measure of overall motivation (Hendijani, 2025a,b; Hendijani et al., 2016; Hendijani and Steel, 2020, 2022). In fact, the amount of time spent on a task is a widely used behavioral measure of motivation in the motivation literature (Baumeister et al., 1998; Deci et al., 1999; Hendijani et al., 2016). When measured during the main experimental session, time-on-task reflects the combined influence of the motivational mechanisms operating throughout the task. Thus, it provides a behavioral measure of **overall motivation**, as it captures the extent to which participants are willing to invest time in task completion (Hendijani et al., 2016; Hendijani and Steel, 2022). Performance is evaluated by the number of correctly recalled words in the memory test.

## 5 Results

### 5.1 Descriptive statistics

To test the research hypotheses, 270 individuals participated in the study. Participants were randomly assigned to one of the nine experimental conditions. Each condition had 30 participants. To estimate the required sample size, we conducted an *a priori* power analysis with the use of G^*^Power software for the two main study variables of reward and choice (Faul et al., 2009). The results indicated that for a power of 0.90, an approximate sample of 235 participants could detect the minimum *f*^2^ effect sizes of 0.045 for choice (three levels: control, no-choice, choice) and 0.055 for reward (three levels: salient, non-salient and control), respectively (Faul et al., 2009). The effect sizes were obtained from prior studies on choice (Patall et al., 2008) and reward (Hendijani and Steel, 2020; 2022; Kim et al., 2022; Weibel et al., 2010).

The participants were 31.48% male and their average age was 22.75 (SD = 4.07). Descriptive statistics for mediating and dependent variables are presented in Table 1. Overall motivation is measured by the amount of time (in minutes) spent on the memory test. The mean and SD values for overall motivation are 18.06 and 0.94, respectively. Performance is calculated by the number of correctly recalled words in the memory test. The mean and SD of performance are 44.73 and 12.98, respectively. Visual attention measures are assessed in milliseconds.

**Table 1:** Descriptive statistics and correlations between mediating and dependent variables.

Variable	Min	Max	Mean (%)	Std Dev. (%)	1	2	3	4
Overall motivation	3.90	51.200	18.060	0.940	–			
Performance	0	60.000	44.728	12.981	0.445^***^	–		
TFF	0	30.190	4.338	3.947	0.726^***^	0.268^***^	–	
TFD	0	1.730	0.228	0.241	0.353^***^	0.143^*^	0.128^*^	–
FC	0	7.210	0.833	0.828	0.368^***^	0.221^***^	0.134^*^	0.924^*^

^*^p < 0.05, ^***^p < 0.001.

TFF, time to first fixation; TFD, total fixation duration; FC, fixation count.

### 5.2 Manipulation check for reward and choice

To check whether the manipulation for reward salience and choice worked, participants answered after the memory test several questions on a seven-point Likert scale (1 = Not at all true, 4 = Somewhat true, 7 = Very true). These questions were similar to the ones used in prior studies (Hendijani and Steel, 2020, 2022). Choice manipulation was checked with four items: (1) “I felt like it was NOT my own choice to take this type of memory test”; (2) “I was NOT given the choice to select the type of memory test”; (3) “I did this activity because I had no choice”; and (4) “I was given the choice to select the type of memory test I wanted to take in this experiment.” The first three items were reversed in calculating the total score for choice manipulation, named *ChoiceMan*. To check whether the choice manipulation was successful, choice and control were regressed on ChoiceMan. The results of the regression analysis were significant. Participants in the Choice (*B* = 1.68, *p* < 0.001) and Control (*B* = 0.51, *p* < 0.001) conditions had significantly higher scores in ChoiceMan compared to participants in the no-choice condition. These results confirm that the manipulation for choice worked out. They indicate that both choice and control conditions perceived higher levels of autonomy and choice compared to the no-choice condition.

Reward salience manipulation was checked with three items: (1) “Money was emphasized a lot in this experiment”; (2) “Monetary reward was a highlighted aspect of this experiment”; and (3) “Monetary reward was mentioned several times in different parts of the experiment.” A variable called *RewardSal* was calculated by taking the average score of these three items. The results of the regression test of Reward type on RewardSal was also significant. Participants in the salient reward condition had significantly higher scores of RewardSal compared to the participants in the non-salient reward condition (*B* = 0.78, *p* < 0.001). Thus, the manipulation for reward salience was successful.

### 5.3 Hypotheses test

To test the hypotheses regarding the effect of choice and reward on overall motivation and performance, two multiple regressions were conducted with overall motivation and performance as the dependent variables. For the choice effect, the two categories of Choice and Control groups were added to the regression model. No-choice condition was considered as the reference group. For the reward effect, Salient and Non-salient reward groups were added to the regression model. The control condition for reward manipulation was considered as the reference group. The results are presented in Table 2.

**Table 2:** Multiple regression results for overall motivation and performance.

Variable	Model I (DV = overall motivation)	Model II (DV = performance)
	*B* (Sig.)	*B* (Sig.)
Constant	20.034 (0.000)	47.636 (0.000)
Control	1.173 (0.322)	1.082 (0.562)
Choice	2.244 (0.056)	1.376 (0.457)
Salient reward	4.265^***^ (0.000)	7.299^***^ (0.000)
Non-salient reward	4.078^***^ (0.001)	7.714^***^ (0.000)
*R* ^2^	0.070	0.076
Adjusted *R*^2^	0.056	0.063
*F* statistic	5.000^***^	5.526^***^
*p*	0.001	0.000
*N*	270	270

^***^p < 0.001.

Model I provides the results of the multiple regression test for overall motivation as the dependent variable. As can be seen, Choice (*B* = 2.244, *p* = 0.056) has no significant effect on overall motivation compared to the no-choice condition. Thus, hypotheses H1a is supported. In addition, salient reward (*B* = 4.265, *p* < 0.001) and non-salient reward (*B* = 4.078, *p* < 0.001) both had significant positive effects on overall motivation. Thus, hypothesis H2a is supported for both salient and non-salient reward types.

**Figure 4:**
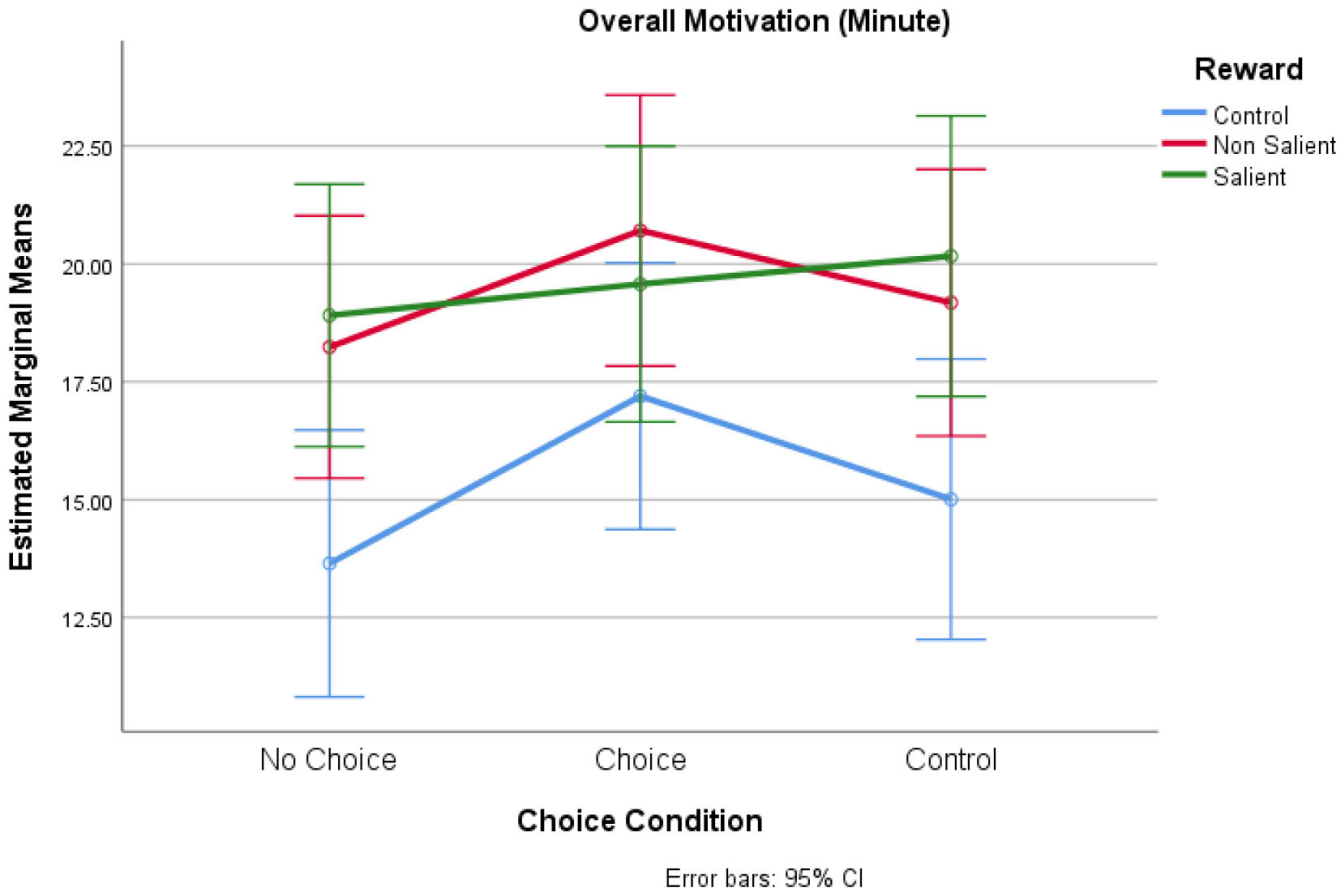
Reward and choice effects on overall motivation.

**Figure 5:**
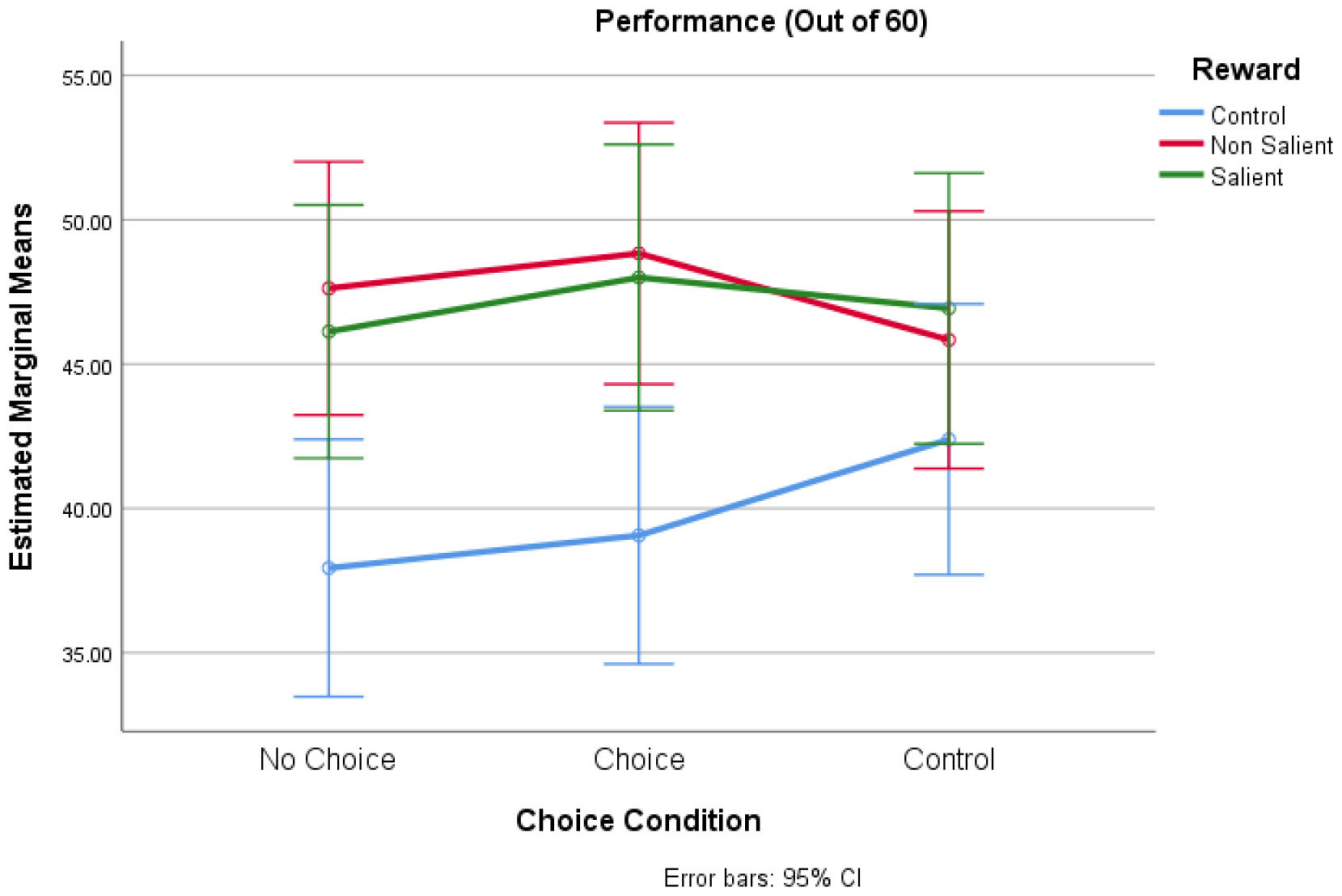
Reward and choice effects on performance.

Model II provides the results of the multiple regression test with performance as the dependent variable. Choice (*B* = 1.376, *p* = 0.457) has no significant effect on performance compared to the no-choice condition. Thus, hypotheses H1b is supported. In addition, salient reward (*B* = 7.299, *p* < 0.001) and non-salient reward (*B* = 7.714, *p* < 0.001) both had significant positive effects on performance. Thus, hypothesis H2b is supported for both salient and non-salient reward types. Figures 4, 5 illustrate the effect of reward and choice on overall motivation and performance, respectively. As can be observed, salient and non-salient rewards have significantly higher effects on overall motivation and performance, compared to the no-reward condition.

### 5.4 Mediation tests

To test the hypotheses related to the mediating effect of visual attention, we conducted mediation tests using PROCESS add-in on SPSS software (Igartua and Hayes, 2021; Preacher et al., 2007). The use of PROCESS add-in for mediation test has several advantages (Shrout and Bolger, 2002). First, it uses a bootstrapping technique that does not require the assumption of normality. Second, it improves statistical power and is able to detect effects even in small and medium sample sizes (Fritz and MacKinnon, 2007). The mediation tests were conducted for overall motivation and performance, separately. To summarize, a direct effect represents the influence of *X* on *Y* that is not transmitted through a mediator (M), while an indirect effect is still the influence of *X* on *Y* but is transmitted through M. The direct and indirect effects collectively represent the observed bivariate association of *X* with *Y*.

### 5.5 Mediation tests on overall motivation.

To test the mediating effect of visual attention on the relationship between salient reward and overall motivation, three mediation tests were conducted with salient reward as the independent variable and Time to First Fixation (TFF), Total Fixation Duration (TFD), and Fixation Count (FC) as the mediating variables, respectively. These measures of visual attention were assessed for the progress/reward box as the area of interest (AOI) related to the reward stimuli. The same procedure was used to test the mediating effects for non-salient rewards. Since the variables in both models were similar, the regression results remained the same. To avoid repetition, we provide the results of the mediation tests for both non-salient and salient rewards only once. Tables 3–5 present these results.

**Table 3:** Overall motivation: mediation effect of time to first fixation (TFF).

TFF regressed on	*B*	SE	Lower 95% CI	Upper 95% CI
Constant	5.044	0.341	4.372	5.716
Salient^**^	1.790	0.587	0.635	2.946
Non-salient^*^	1.528	0.579	0.389	2.668
Control	0.476	0.581	−0.668	1.621
Choice	0.382	0.576	−0.752	1.516
*R^2^* (%) = 4.25 (*p* < 0.05).				
**Overall motivation regressed on**	***B***	**SE**	**Lower 95%**	**Upper 95%**
Constant	12.711	0.664	11.404	14.019
TFF^***^	1.465	0.089	1.290	1.640
Salient	1.608	0.859	−0.084	3.299
Non-salient^*^	1.879	0.844	0.219	3.540
Control	0.496	0.837	−1.152	2.145
Choice	1.796	0.829	0.163	3.429
*R^2^* (%) = 54.47 (p < 0.001).				

^*^p < 0.05, ^**^p < 0.01, ^***^p < 0.001.

**Table 4:** Overall motivation: mediation effect of total fixation duration (TFD).

TFD regressed on	*B*	SE	Lower 95% CI	Upper 95% CI
Constant	0.240	0.020	0.200	0.281
Salient^**^	0.096	0.035	0.026	0.166
Non-salient^**^	0.101	0.035	0.032	0.170
Control^*^	−0.073	0.035	−0.142	−0.004
Choice	−0.038	−0.035	−0.107	0.030
*R^2^* (%) = 5.26 (*p* < 0.01).				
**Overall motivation regressed on**	***B***	**SE**	**Lower 95%**	**Upper 95%**
Constant	17.319	0.803	15.738	18.900
TFD^***^	11.329	1.143	7.483	15.175
Salient^**^	3.136	1.143	0.885	5.386
Non-salient^*^	2.891	1.133	0.661	5.122
Control	2.003	1.125	−0.213	4.219
Choice^*^	2.719	1.112	0.530	4.909
*R^2^* (%) = 54.47 (p < 0.001).				

^*^p < 0.05, ^**^p < 0.01, ^***^p < 0.001.

**Table 5:** Overall motivation: mediation effect of fixation count (FC).

FC regressed on	*B*	SE	Lower 95% CI	Upper 95% CI
Constant	0.887	0.070	0.794	1.025
Salient^*^	0.294	0.121	0.055	0.533
Non-salient^***^	0.428	0.120	0.192	0.664
Control	−0.232	0.120	−0.468	0.004
Choice	0.124	0.119	−0.359	0.111
*R^2^* (%) = 5.97 (*p* < 0.01).				
**Overall motivation regressed on**	***B***	**SE**	**Lower 95%**	**Upper 95%**
Constant	16.981	0.820	15.366	18.595
FC^***^	3.449	0.567	2.333	4.566
Salient^**^	3.209	1.133	0.977	5.441
Non-salient^*^	2.561	1.135	0.326	4.796
Control	1.973	1.118	−0.227	4.174
Choice^*^	2.714	1.105	0.538	4.891
R^2^ (%) = 18.31 (p < 0.001).				

^*^p < 0.05, ^**^p < 0.01, ^***^p < 0.001.

Table 3 provides the results for the mediating effect of TFF. As can be seen in the second regression analysis, the direct effect of salient reward on overall motivation is not significant (Effect = 1.608, 95% CI [−0.084, 3.300]). The indirect effect of salient reward on overall motivation through the mediating effect of TFF is significant (Effect = 2.622, 95% CI [1.096, 4.160]). Thus, TFF fully mediates the effect of salient reward on overall motivation. For non-salient reward, both the direct (Effect = 1.879, 95% CI [0.219, 3.540]) and indirect (Effect = 2.239, 95% CI [0.854, 3.920]) effects are significant. Thus, TFF partially mediates the effect of non-salient reward on overall motivation. In total, TFF mediates the effect of salient and non-salient rewards on overall motivation. Therefore, H3a is supported for TFF as one of the measures of visual attention.

Table 4 presents the results for the mediating effect of TFD. The direct (Effect = 3.136, 95% CI [0.885, 5.386]) and indirect (Effect = 1.087, 95% CI [0.395, 1.956]) effects of salient reward on overall motivation through the mediating effect of TFD are significant. Similarly, the direct (Effect = 2.891, 95% CI [0.661, 5.122]) and indirect (Effect = 1.145, 95% CI [0.510, 1.863]) of non-salient reward on overall motivation through the mediating effect of TFF are significant. Thus, TFD partially mediates the effect of salient and non-salient reward on overall motivation and H3a is supported for TFD as one of the measures of visual attention.

Table 5 presents the results for the mediating effect of FC. The direct (Effect = 3.209, 95% CI [0.977, 5.441]) and indirect (Effect = 1.014, 95% CI [0.343, 1.934]) effects of salient reward on overall motivation through the mediating effect of FC are significant. Similarly, the direct (Effect = 2.561, 95% CI [0.326, 4.796]) and indirect (Effect = 1.476, 95% CI [0.739, 2.249]) of non-salient reward on overall motivation through the mediating effect of FC are significant. Thus, FC partially mediates the effect of salient and non-salient reward on overall motivation and H3a is supported for FC as one of the measures of visual attention.

### 5.6 Mediation tests on performance

To test the mediating effect of visual attention on the relationship between salient reward and non-salient reward on performance, we used the same procedure as we did for overall motivation. This time performance was added to the mediation models as the dependent variable. These results are presented in Tables 6–8.

**Table 6:** Performance: mediation effect of time to first fixation (TFF).

TFF regressed on	*B*	SE	Lower 95% CI	Upper 95% CI
Constant	5.044	0.341	4.373	5.716
Salient^**^	1.790	0.587	0.635	2.946
Non-salient^**^	1.528	0.579	0.389	2.668
Control	0.476	0.581	−0.668	1.621
Choice	0.382	0.576	−0.752	1.516
*R^2^* (%) = 4.25 (*p* < 0.05).				
**Performance regressed on**	***B***	**SE**	**Lower 95%**	**Upper 95%**
Constant	44.046	1.460	41.172	46.921
TFF^***^	0.732	0.195	0.348	1.116
Salient^**^	6.008	1.889	2.290	9.726
Non-salient^***^	6.818	1.854	3.168	10.469
Control	0.847	1.840	−2.776	4.471
Choice	1.106	1.823	−2.483	4.694
R^2^ (%) = 12.58 (p < 0.001).				

^*^p < 0.05, ^**^p < 0.01, ^***^p < 0.001.

**Table 7:** Performance: mediation effect of total fixation duration (TFD).

TFD regressed on	*B*	SE	Lower 95% CI	Upper 95% CI
Constant	0.240	0.020	0.200	0.281
Salient^**^	0.096	0.035	0.026	0.166
Non-salient^**^	0.101	0.035	0.032	0.170
Control^*^	−0.073	0.035	−0.142	−0.004
Choice	−0.038	−0.035	−0.107	0.030
*R^2^* (%) = 5.26 (*p* < 0.01).				
**Performance regressed on**	***B***	**SE**	**Lower 95%**	**Upper 95%**
Constant	46.330	1.340	43.692	48.968
TFD	5.408	3.260	−1.010	11.826
Salient^***^	6.822	1.907	3.067	10.577
Non-salient^***^	7.209	1.890	3.488	10.930
Control	1.478	1.878	−2.219	5.175
Choice^*^	1.541	1.855	−2.112	5.195
*R^2^* (%) = 8.62 (p < 0.001).				

^*^p < 0.05, ^**^p < 0.01, ^***^p < 0.001.

**Table 8:** Performance: mediation effect of fixation count (FC).

FC regressed on	*B*	SE	Lower 95% CI	Upper 95% CI
Constant	0.887	0.070	0.749	1.025
Salient^*^	0.294	0.121	0.055	0.533
Non-salient^***^	0.428	0.120	0.192	0.664
Control	0.232	0.120	−0.468	0.004
Choice	−0.124	0.119	−0.359	0.111
*R^2^* (%) = 5.97 (*p* < 0.01).				
**Performance regressed on**	***B***	**SE**	**Lower 95%**	**Upper 95%**
Constant	45.136	1.361	42.457	47.815
FC^**^	2.810	0.941	0.958	4.662
Salient^***^	6.515	1.880	2.812	10.217
Non-salient^***^	6.553	1.883	2.845	10.262
Control	1.734	1.854	−1.917	5.385
Choice	1.684	1.834	−1.927	5.295
*R^2^* (%) = 10.68 (*p* < 0.001).				

^*^p < 0.05, ^**^p < 0.01, ^***^p < 0.001.

Table 6 presents the results for the mediating effect of TFF. The direct (Effect = 6.008, 95% CI [2.290, 9.726]) and indirect (Effect = 1.310, 95% CI [0.102, 2.805]) effects of salient reward on performance through the mediating effect of TFF are significant. Similarly, the direct (Effect = 6.818, 95% CI [3.168, 10.469]) and indirect (Effect = 1.118, 95% CI [0.042, 2.298]) of non-salient reward on performance through the mediating effect of TFF are significant. Thus, TFF partially mediates the effect of salient and non-salient reward on performance and H3b is supported for TFF as one of the measures of visual attention.

Table 7 presents the results for the mediating effect of TFD. The direct effect (Effect = 6.822, 95% CI [3.067, 10.577]) of salient reward on performance is significant, but the indirect effect (Effect = 0.519, 95% CI [−0.243, 1.295]) through TDF is not significant. Similarly, the direct effect (Effect = 7.209, 95% CI [3.488, 10.930]) of non-salient reward on performance is significant, but the indirect effect (Effect = 0.547, 95% CI [−0.204, 1.596]) through TDF is not significant. Thus, TFD does not mediate the effect of salient and non-salient reward on performance and H3b is not supported for TFD.

Table 8 presents the results for the mediating effect of FC. The direct (Effect = 6.515, 95% CI [2.812, 10.217]) and indirect (Effect = 0.826, 95% CI [0.217, 1.765]) effects of salient reward are significant. Similarly, the direct (Effect = 6.553, 95% CI [2.845, 10.262]) and indirect (Effect = 1.202, 95% CI [0.417, 2.233]) effects of non-salient reward on performance are significant. Thus, FC partially mediates the effect of salient and non-salient reward on performance and H3b is supported for FC as one of the measures of visual attention.

In total, the results of the mediation tests provide support for the mediating effect of all three measures of visual attention, including TFF, TFD, and FC on overall motivation and two measures of visual attention, including TFF and FC on performance. Thus, Hypotheses H3a and H3b are supported for most measures of visual attention.

## 6 Conclusion

Two motivational mechanisms that have been widely used to increase motivation and performance are rewards (Garbers and Konradt, 2014) and choice (Patall et al., 2010). Despite their widespread use, there is still a longstanding debate regarding their effects (Hendijani and Steel, 2020, 2022), with major motivational theories providing opposing predictions regarding their importance and effect on motivation and performance. Behavioral theories in psychology and classic economics consider performance-contingent reward as the major type of motivational mechanism that can reinforce motivation and performance (Eisenberger and Cameron, 1996; Skinner, 1953; Vroom, 1964). On the other hand, cognitive evaluation and self-determination theories give a high weight to autonomy and consider it as one of the main pillars of intrinsic motivation and their use results in adaptive behavioral outcomes (Ryan and Deci, 2000). According to these theories, choice is an embodiment of autonomy. Hence, its provision reinforces intrinsic motivation. In contrast, performance-contingent rewards are controlling and can undermine feelings of autonomy and intrinsic motivation. Previous empirical studies have found that contextual and task-related factors can influence the effect of these motivational mechanisms on overall motivation and performance (Amabile et al., 1976; Koh, 2020; Kuvaas et al., 2016; Thomas and Mueller, 2017). MCT tries to resolve this debate by giving equal importance to these two motivational mechanisms and adding the role of context in their interrelationship and effect on overall motivation and performance (Hendijani and Steel, 2024).

Built upon MCT, this study examines the role of performance-contingent reward and choice on overall motivation and performance. In addition to examining the direct effect of reward and choice, it evaluates the mediating effect of visual attention toward the reward-related information. This allows us to integrate the cognitive and behavioral effects of these motivational mechanisms and sheds light on the cognitive mechanisms that underlie the behavioral outcomes of overall motivation and performance.

Furthermore, this study is conducted in a controlling context to highlight the role of context as a determining factor on the relationship between motivational mechanisms and behavioral outcomes. To create such a context, several characteristics are incorporated in the task and the environment. First, the experimental task is an attention-demanding memory test. Prior studies have shown that attention demand is one of the elements that can negatively influence feelings of autonomy (Hendijani and Steel, 2022). In addition to the attention demand, other task characteristics and contextual elements contributed to the controlling nature of the task environment. These include: presenting a list of pre-ordered and pre-determined words, not allowing participants to go back and revisit the previously presented words, having a time limit of 1-h for the test, and automatically ending the test after the 1-h time interval.

The study produced several major results which contribute to the existing body of literature. First, choice has no significant effect on overall motivation and performance. The insignificant effect of choice can be attributed to the controlling nature of the context. The demanding nature of the memory test along with other autonomy thwarting contextual elements created a type of environment, which attenuated the motivational advantages of choice. This result is consistent with MCT's prediction that choice loses its motivational effect when administered in a controlling context (Hendijani and Steel, 2024; Xue et al., 2023).

Second, performance-contingent rewards both in salient and non-salient types improve overall motivation and performance. These provide further support for MCT's predictions regarding the positive effect of performance-contingent rewards on overall motivation and performance in a controlling context. In addition, they match the findings of previous empirical studies (Hendijani and Steel, 2022).

In addition to these behavioral aspects, this study examined the role of visual attention toward the reward-related stimuli as an underlying cognitive mechanism on the effect of performance-contingent rewards on overall motivation and performance. The results supported the mediating effect of visual attention. These results provide deep insights on how performance-contingent rewards can influence overall motivation and performance by increasing attention toward the reward-related stimuli. They provide further support for the important role of congruence between the motivational mechanism and the context as explicated by MCT. According to these results, performance-contingent rewards can match the attention-demanding nature of the task and environment. Such a congruence results in higher visual attention toward the reward-related stimuli and in turn, improve overall motivation and performance. Previous studies on the effect of reward on visual attention have produced similar results. Rewards, especially performance-contingent ones shift the individuals' attention toward the reward-related stimuli (Chelazzi et al., 2013; Failing and Theeuwes, 2018; Hickey et al., 2010; Stănişor et al., 2013).

This study has several advantages. First, it takes a new approach based on MCT to address a long-standing debate regarding the effect of two major motivational mechanisms of reward and choice. MCT's contextualistic view and its emphasis on congruence between motivational mechanisms and the context help in resolving the contradictory results that were found in previous theoretical and empirical studies (Hendijani and Steel, 2024; Pekrun, 2024). The congruence effect has been viewed in other studies as well. For example, the results of a meta-analytical review indicates that the provision of rationale as a motivational mechanism is most effective when the language or the context is non-controlling (Steingut et al., 2017). Second, the study examines the effects of these mechanisms both from cognitive and behavioral perspectives. This approach has not been taken in the past literature. In line with the calls for a more integrative approach in motivation science (King and Fryer, 2024; Pekrun, 2024), it bridges the gap between the psychophysiological and psychological studies on motivation and performance. The use of visual attention measures allows us to get a deep understanding of the cognitive mechanisms underlying motivation (Moore and Zirnsak, 2017; Ungerleider, 2000). In fact, these measures provide worthy information regarding the perceptual and visual attention toward the reward-related information and its influence on motivation and performance. It is important to note that the eye-tracking measures assessed attention toward the reward/test progress information not toward the presented words or the whole screen. The areas of interest were limited to the bottom right hand of the screen where the reward information or test progress information was presented. These results indicate that the individuals in the reward conditions pay more visual attention toward the reward information presented on the screen and this intense attention increases overall motivation and performance compared to the control condition.

Another interesting result of eye-tracking measurement relates to the comparison between non-salient and control conditions. In fact, participants in the non-salient reward condition saw the same thing (i.e., test progress) as the participants in the control condition. However, these participants paid more visual attention toward this part of the screen and the increased visual attention contributed to the escalation of overall motivation and performance. This can be interpreted that participants in the non-salient reward condition perceived test progress as an implicit signal for the potential reward even though it was not explicitly mentioned as was done in the salient reward condition.

As a whole, the eye-tracking results indicate that the provision of separate information regarding reward on the screen both implicitly (by showing test progress) or explicitly (by showing potential earning) could be beneficial not distracting as one might presume due to the highly attention demanding nature of the memory test. The increase in visual attention in turn improves overall motivation and performance. These results strengthen the predictions of MCT at a deeper cognitive level rather than the mere behavioral one.

Finally, this study sheds light on the context-dependent nature of motivation. By focusing on a task context that is inherently controlling, the study sheds light on how context can influence the way motivational mechanisms are perceived and utilized by individuals toward obtaining the related performance outcomes.

This study has some limitations. First, the context in this study is controlling. Future studies should examine these effects in autonomy-supportive contexts as well. Such contexts can be created by producing a self-directed task where the participants feels high levels of autonomy and control over different aspects of the task, including task design, order, time, and approach to addressing and solving the task at hand (Gureckis and Markant, 2012a,b; Sobel and Kushnir, 2006). Second, the task is an attention-demanding memory test. It is recommended to use other task types in future studies to increase the difference and commonalities of the results based on the task type and nature. Third, this study used visual attention as a cognitive mechanism that underlies behavioral outcomes of overall motivation and performance. It is suggested that future studies examine other cognitive mechanisms such as brain signals to get a deeper understanding of the cognitive mechanisms that underlie the effect of these motivational mechanisms on overall motivation and performance (Baier-Mosch et al., 2024; Cromwell et al., 2020; Michaelsen and Esch, 2021; Simpson and Balsam, 2016). Fourth, this study uses time spent on the task as a single measure for overall motivation. Since time spent on the task is directly linked with performance, future studies should use other measures for overall motivation to validate and increase the generalizability of the results regarding the relationship between reward, overall motivation, and performance. In addition, it would be worthwhile to design and conduct an experiment with a yoked control no-reward group that has an identical timing with the salient reward group. This can help distinguish and separate the effect of reward from the effect of time spent on the task on performance.

## Data Availability

The raw data supporting the conclusions of this article will be made available by the authors, without undue reservation.
